# Geographic-Related Differences of Pituitary Adenomas Hormone Profile: Analysis of Two Groups Coming from Southeastern and Eastern Europe

**DOI:** 10.1155/2015/192094

**Published:** 2015-05-11

**Authors:** Anca Maria Cimpean, Eugen Melnic, Bogdan Bălinişteanu, Ana Corlan, Mihail Coculescu, Sergiu Rusu, Marius Raica

**Affiliations:** ^1^Department of Microscopic Morphology/Histology, Angiogenesis Research Center, “Victor Babes” University of Medicine and Pharmacy, Timisoara, Romania; ^2^Department of Pathology, “Nicolae Testemiţanu” University of Medicine and Pharmacy, Chișinău, Moldova; ^3^Department of Endocrinology, “Victor Babes” University of Medicine and Pharmacy, Timisoara, Romania; ^4^National Institute of Endocrinology I. C. Parhon, “Carol Davila” University of Medicine and Pharmacy, Bucharest, Romania

## Abstract

We compared the immunoprofile of pituitary adenomas from Romania and Moldova. One hundred and eighty cases coming from Romania (94 cases, group 1) and Moldova (86 cases, group 2) were assessed by immunohistochemistry regarding all six basic hormones expressed in pituitary adenomas. Specific differences and similarities were found and stated for both groups. In group 1, 70% of cases were pituitary adenomas positive for one hormone, 13% were plurihormonal, while 17% were negative. In group 2, 50,3% of the cases expressed only one hormone and 12,5% were negative for all hormones. The highest difference was observed for plurihormonal adenomas, found in about 37,2% of cases for group 2 (2.86 times higher for group 2 compared with group 1). A higher incidence of GH-secreting adenomas characterized group “1,” while group “2” had the highest percent of LH-secreting adenomas, 55% of cases being positive. Triple association was noticed in 4.25% of cases of group 1 and in 8,13% out of total cases, from group 2. Four-hormone association was found only in group 2, noticed in 15,56% of the cases. The present paper highlights strong evidences of a particular and different immunoprofile of pituitary adenomas coming from Romania and Moldova.

## 1. Introduction

Pituitary adenomas are some of the most frequent intracranial tumors [1], being the most common tumors of the sellar region. Sometimes, their unusual clinical behavior at different ages [2, 3] and hormone immunoprofile heterogeneity [4] make the establishment of a proper diagnosis and appropriate therapeutic options difficult. Pituitary adenoma heterogeneity was also stated at genetic level [5]. Genetic mutations in pituitary tumors are found to be associated with a high incidence of other tumor types such as intracranial tumors [6], Carney syndrome [7, 8], or multiple endocrine neoplasia type 1 [9]. Clinical, immunohistochemical, or genetic questionable results found in the literature are not the only controversies regarding pituitary tumors.

Epidemiological data on pituitary adenomas could help to optimize the allocation of human resources for care of such patients and also improve the accuracy of diagnosis for some neglected geographical regions. Epidemiologic data refer to the prevalence of pituitary tumors and are usually reported for big countries. Pituitary tumor prevalence varies from 19 to 28 cases per 100,000, in the UK, to 94 cases per 100,000 in Belgium, or with an incidence of 0.4 to 8.2 per 100,000 per year [10–12]. A recently published paper regarding descriptive epidemiology of pituitary adenomas in the United States stratified the incidence of pituitary tumors according to the age, sex, race, or subregions. Significant differences were reported regarding pituitary adenomas incidence between different racial groups from the USA [13]. Epidemiologic data about pituitary adenomas in Europe are very scarce, even for the countries with a highly developed health system such as Switzerland or Austria, where registry regarding brain tumors (including pituitary adenomas) is a relatively new topic [14, 15]. For small countries, especially for those of Eastern or Central Europe such statistical data are quite recent such as for Romania [16] or absent such as for Moldova.

Statistical and epidemiological data concerning hormone profile differences among populational groups are scattered, especially stated for prolactinomas [17–19], neglecting other pituitary adenoma types. The studies mentioned above covered a well-delineated area usually comprising one country or one subregion. No comparative assessment of pituitary adenomas hormone profile has been done before between Romanian and Moldavian groups of patients.

The present study is the first stage of a comparative assessment of pituitary adenomas derived from two different geographical regions from Eastern and Southeastern Europe in order to identify potential specific geographical and epidemiological factors influencing the development of pituitary adenomas in these regions of Europe. Thus, the present paper is focused on a comparative study of immunohistochemically assessed pituitary adenomas hormone profiles from the two mentioned regions, for the identification of specific similarities and differences between Romanian and Moldavian groups of intracranial masses previously diagnosed as pituitary adenomas. Further studies will correlate the findings regarding hormone profiles differences with other clinical and epidemiological data.

## 2. Material and Methods

For our purpose, a retrospective study has been designed by randomly choosing and including a total number of 180 cases organized in two distinct groups. The collected specimens covered the same period of time for both groups, selected cases being diagnosed between 2009 and 2012. Cases were included in the research flow by the selection of paraffin blocks and their histopathological evaluation. Because of its basic research type, the present study was exclusively focused on identification of hormone immunoprofile differences between two groups and based on these differences to highlight epidemiological and/or geographical factors with a possible influence on pituitary adenomas development in future studies.

The first group, named “1,” included 94 cases of pituitary adenomas coming from the Department of Endocrinology of “Carol Davila” University of Medicine and Pharmacy, Bucharest, Romania, while the second group, named “2,” was organized by selection of 86 cases of pituitary adenomas from the Department of Pathology of “Nicolae Testemiţanu” University of Medicine and Pharmacy, Chişinău, Moldova. Most of the cases included in group “1” were collected from the eastern and southern part of Romania, while cases included in group “2” were collected from all Moldova regions. Each diagnosis was certified by clinical, biological, and imagistic data. Specimens were collected by open surgery or by transsphenoidal approach and were processed in a similar manner following routine standard protocols for paraffin embedding, carefully handled by specialized histotechnicians. Biopsies were fixed in 10% buffered formalin for 48 hours and paraffin-embedded. All paraffin blocks from Bucharest and Chişinău were then sent to the Angiogenesis Research Center, Timisoara, where the histopathological and immunohistochemical techniques were developed.

Three-micrometer-thick serial sections were obtained from each paraffin block. Histopathology was assessed on hematoxylin and eosin stained slides. Based on morphologic evaluation, additional sections from each case were selected to perform hormone profile of pituitary adenomas. Specimens for immunohistochemistry were also carefully selected after performing a vimentin (clone V9) control staining (to check whether the primary processing of tissue biopsies was properly done). There were performed immunohistochemical procedures to highlight all six pituitary hormones such as growth hormone (GH, polyclonal rabbit anti-human, dilution 1 : 300), prolactin (PRL, polyclonal rabbit anti-human, dilution 1 : 250), adrenocorticotropin (ACTH, monoclonal mouse anti-human, clone 02A3, dilution 1 : 50), thyroid stimulating hormone (TSH, monoclonal mouse anti-human, clone 0042, dilution 1 : 50), luteinizing hormone (LH, monoclonal mouse anti-human, clone C93, dilution 1 : 50), and follicle stimulating hormone (FSH, monoclonal mouse anti-human, clone C10, dilution 1 : 50). All primary antibodies were supplied by DakoCytomation, Carpinteria, USA. Thirty-minute incubation with primary antibodies at room temperature was followed by the use of Polymer Refine Detection System (Novocastra, Newcastle, United Kingdom). All cases were evaluated by using as positive control normal pituitary tissue samples assessed for previously mentioned hormones expression. As negative control slides, we also used normal human pituitary gland tissue (obtained by autopsy) where we omitted to add the primary antibodies.

The entire immunohistochemical procedure was performed with Bond Max Automated System (Leica Microsystems, Newcastle, UK). Microscopic evaluation was performed by three independent observers using Nikon Eclipse E600 (Nikon Corporation, Tokyo, Japan). Images were captured and processed with Lucia G software system. The presence of more than 10% of hormone immunopositive cells indicated a hormone-producing tumor [20]. The tumors with strong coexpression of GH/PRL (>50% of cells) were considered pituitary tumors cosecreting GH and PRL. An overall and specific comparative assessment of hormone profile was performed between groups “1” and “2.”

Histopathology, immunohistochemistry, and data interpretation were done at the Department of Microscopic Morphology/Histology of “Victor Babes” University of Medicine and Pharmacy, Timisoara, Romania. Statistical analysis was performed by using SPSS software version 17.0 package.

The local research ethics committee approved the study protocol, and informed consent was obtained from all subjects according to the* World Medical Association Declaration of Helsinki*.

## 3. Results

**Figure 1:**
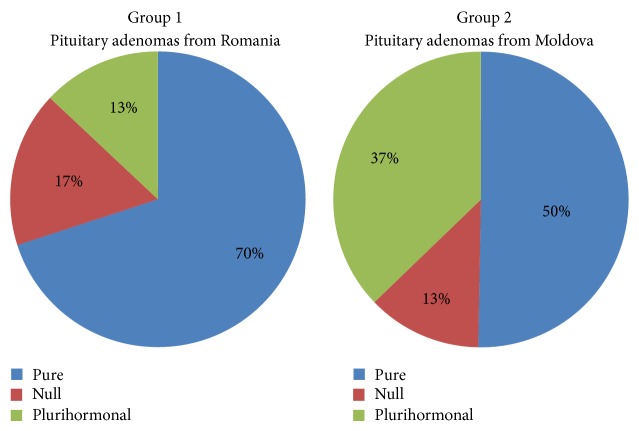
Comparative assessment of both groups regarding types of pituitary adenoma based on their immunoprofile. Percentage of pure, null cell adenomas and plurihormonal adenomas between group 1 (pituitary adenomas from Romania) and group 2 (pituitary adenomas from Moldova). The highest difference between these two groups was noticed for plurihormonal adenomas.

Overall assessment of both groups 1 and 2 showed differences regarding hormone profile of pituitary adenomas from Romania and Moldova. For group 1, 70% of cases were pituitary adenomas positive for one hormone, 13% were plurihormonal adenomas, while 17% were negative for all six hormone types. Compared with group 1, several differences were counted for group 2. From the cases of group 2, 50,3% expressed only one hormone and 12,5% were negative for all hormones. The highest difference was observed for plurihormonal adenomas, found in about 37,2% of cases for group 2 (2.86 times higher for group 2 compared with group 1) (Figure 1).

Hormone specific assessment demonstrated a higher incidence of GH-secreting adenomas in group “1” compared with group “2.” About 46% of 94 cases of group “1” were represented by GH-secreting adenomas compared with group “2” where we found only 25% of GH-secreting adenomas. PRL-secreting adenomas values were also different for the two groups. Twenty-three percent of the cases from group “1” were PRL-positive, while for group 2 only 10% of cases showed positive immunoreaction for PRL. No pure TSH-secreting adenomas were observed either for group “1” or for group “2.” The incidence of ACTH-secreting adenomas from group “1” was quite similar to that found for group “2,” although a small difference of two percent was present between these groups (6% for group “1” versus 5% for group “2”). This was the highest overlapping regarding the incidence of positive cases between the two groups included in the present study.

**Figure 2:**
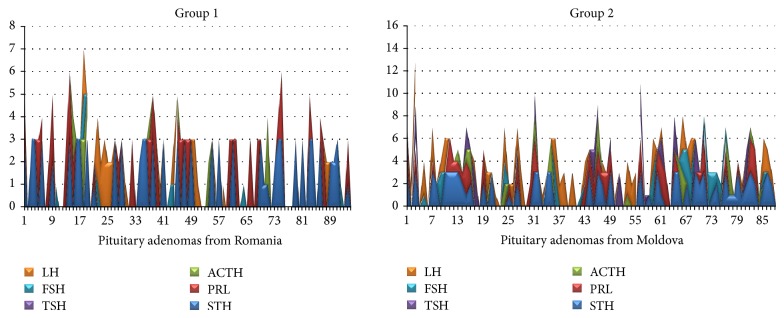
Mapping of pituitary adenomas hormone profiles was comparatively highlighted by overlapping charts for all six hormone types. The differential distribution and density of cases positive for different kinds of hormones between the two groups were shown.

Regarding FSH, 7.7% of the cases from group “1” and 5% from group “2” were immunopositive. The highest difference between the two groups was registered for LH-secreting adenomas. Group 2 had the highest value of LH-secreting adenomas; 55% of cases were found positive (Figure 2).

**Figure 3:**
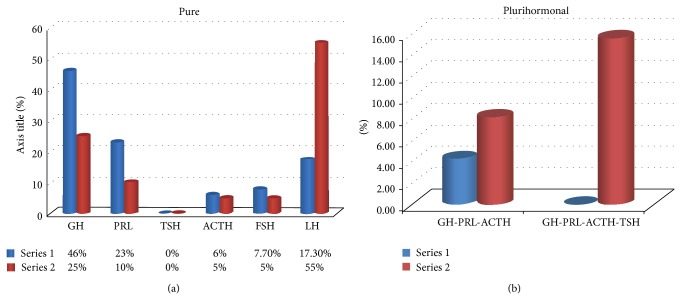
Graphic distribution of pure pituitary adenomas (a) and plurihormonal adenomas (b) between the two groups. The particular high number of GH- and LH-secreting pure adenomas (a) and plurihormonal adenomas with triple and quadruple hormone (b) associations was specific for group 2 (pituitary adenomas from Moldova), compared with group 1 (pituitary adenomas from Romania).

Plurihormonal adenomas were split in two subgroups which were separately evaluated. The most well-known association found in pituitary adenomas assessment, between GH and PRL, was present in 10.36% of the cases from group 1 and in 13.63% of the cases from group 2. Triple hormone associations were noticed in both groups, but with differences regarding their percentage and hormone types. If group 1 showed triple association in 4.25% of cases, for group 2, this association has been shown in 8,13% of the cases. GH-PRL-ACTH association has been found as predominant for both groups. The association between four hormones was specific for group 2 only, being found in 15,56% of cases (Figure 3). In comparison with triple association, TSH was the hormone added to this quadruple association.

## 4. Discussion

Data about geographically related heterogeneity of pituitary tumors were previously published in relation with genetic variability especially observed for familiar pituitary adenomas [21]. Few population-based studies of the epidemiology of pituitary adenomas have been reported due to the difficulties in undertaking such studies which require close scrutiny of patients for inclusion and accurate definition of study populations. The largest studies regarding pituitary adenomas regional differences were developed by Clayton, who reported an overall prevalence of 190–280 cases/million, of whom 31.6–35.7% had prolactinomas, 32.1–36.8% had nonsecreting tumors, 21.1–21.4% had somatotropinomas, and 10.5–10.7% had Cushing's disease [22]. Our study highlighted differences between groups “1” and “2” regarding types of pituitary adenomas and the values obtained in the present paper are different compared with those previously reported by Clayton. GH-secreting adenomas cases were twice higher for group 1 compared with Clayton's study, while, for group 2, the percentage of GH-secreting adenomas was similar to that reported by Clayton. In another study performed in the Province of Liege, Belgium [11], GH-secreting adenomas were found in about 13,2% of cases, the lowest value reported for such type of pituitary adenomas in the literature.

Regarding PRL-secreting adenomas, both groups 1 and 2 had a lower number of cases compared with previous similar populational studies [11, 23, 24]. This may derive from the well-known fact that the majority of patients with prolactinomas are not indicated for neurosurgery but are treated with dopamine agonists. The present study lacks the assessment of treatment of patients with prolactinomas and this could be considered as a limitation of the present study. However, we analysed the hormone profiles of retrospective cases and most probably those cases with prolactinomas which underwent surgery had well-supported reasons such as being probably dopamine agonist resistant prolactinomas or having nonspecific clinical signs and being characterized as prolactinomas by their immunoprofile exclusively. If Ezzat and coworkers reported a high variability of PRL-secreting adenomas percentage between 25 and 41% of cases [24], our study reported a variability ranged between 10% (found in group 2) and 23% (for group 1). Group 2 was characterized by the lowest value of PRL-secreting adenomas reported until now compared with epidemiological studies about pituitary adenomas, recently published in the literature [17]. This could be considered a particular feature of pituitary adenomas specific for Moldova. Even for group 1, the number of PRL-secreting adenomas was lower as compared with previous reports. This being the first comparative observational cross-sectional study including countries from Southeastern and Eastern Europe, further studies need to develop for elucidation of etiological factors influencing the lower number of PRL-secreting adenomas in these regions. Ciccarelli and coworkers reported the variability of pituitary adenomas with age and sex but not with geographical regions [19]. Isolated data regarding geographical variability of prolactinomas percentage were recently reported by Pereira-Lima and coworkers, who related prolactinomas with body weight variability in different regions of Brazil [25].

The lowest number of pituitary adenomas epidemiological studies refers to the ACTH-secreting adenomas [26]. Most of them reported statistical data about clinical, etiological, and therapeutic response variability [27, 28], and few of them focused on geographical distribution and regional differences. Clayton et al. reported about 10% of ACTH-secreting adenomas in their populational study compared with Daly similar study for Liege region where the percentage of ACTH-secreting adenomas has been reported to be about 6% of cases. Both groups included in our study had a percentage of ACTH-secreting adenomas ranged between 5 and 6%, overlapping with Daly study results but different from Clayton's results.

Most of the nonfunctioning pituitary adenomas are gonadotroph-producing adenomas [29]. They comprise about 30 to 35% of the pituitary tumors in most series [30]. There are few data regarding percentage of LH-positive pituitary adenomas because of their assessment together with FSH-secreting adenomas. LH-secreting adenomas were three times more frequently observed in group 2 compared with group 1. To the best of our knowledge, the percentage of LH-secreting adenomas (55%) found in group 2 seems to be the highest value reported until now in the literature.

The percentage of nonsecreting pituitary adenomas ranged between 12,5% (for group 2) and 17% for group 1 in our study. These values were overlapped with data published by Dayl but were lower compared with results reported by Clayton in the already mentioned study.

Plurihormonal adenomas reported in the literature showing positivity for at least three markers assessed in the present study usually contained TSH-positive cells [31], but pituitary adenomas secreting TSH exclusively were rarely found and published as case report in the literature [32–34]. In contrast with these, Yamada et al. recently reported the increase of TSH-producing adenomas over the last five years but this is a single-center study of 90 cases [35]. Most of the plurihormonal associations found in the present study were characterized by the presence of GH-PRL-ACTH, contrasting with literature data which reported the presence of TSH in most of these associations. Although TSH was present, it was a fourth hormone added in about 15.56% of quadruple association found exclusively in group 2 in the present study.

## 5. Conclusions

The present paper highlights strong evidences of a particular and specific immunoprofile of pituitary adenomas coming from two different regions of Eastern Europe. Pituitary adenomas from Romania have an immunoprofile which overlapped in part with the immunoprofile described for pituitary adenomas from other parts of Europe. This study described for the first time the immunoprofile of pituitary adenomas coming from Moldova and by comparison with those from the southern and eastern part of Romania they have a particular immunoprofile characterized by a decreased number of prolactinomas and an increased number of LH-secreting adenomas. Triple or quadruple associations specifically found with a higher frequency for Moldavian group completed the profile of pituitary adenomas. Differences stated in the present paper raise several questions about the presence of regional specific epidemiologic or etiologic factors able to influence the immunoprofile of pituitary adenomas.
